# Nationwide Survey of Pulmonary Physical Rehabilitation for Neonatal Respiratory Distress Syndrome in Very‐Low‐Birth‐Weight Infants in Japan

**DOI:** 10.1111/jpc.70193

**Published:** 2025-09-09

**Authors:** Yuto Ogata, Ryutaro Matsugaki, Manami Zaizen, Keiji Muramatu, Shinya Matsuda, Shutaro Suga, Satoru Saeki

**Affiliations:** ^1^ Department of Rehabilitation University Hospital of Occupational and Environmental Health Kitakyushu Yahatanishi‐ku Japan; ^2^ Department of Work Systems and Health Institute of Industry Ecological Sciences, University of Occupational and Environmental Health Kitakyushu Yahatanishi‐ku Japan; ^3^ Department of Preventive Medicine and Community Health School of Medicine, University of Occupational and Environmental Health Kitakyushu Yahatanishi‐ku Japan; ^4^ Department of Pediatrics University of Occupational and Environmental Health Kitakyushu Yahatanishi‐ku Japan; ^5^ Department of Rehabilitation Medicine University of Occupational and Environmental Health Kitakyushu Yahatanishi‐ku Japan

**Keywords:** diagnosis procedure combination, Japan, neonatal respiratory distress syndrome, pulmonary physical rehabilitation, very low‐birth‐weight infants

## Abstract

**Aim:**

This study aimed to investigate the implementation rate and timing of pulmonary physical rehabilitation for very low‐birth‐weight infants (VLBWI) with respiratory distress syndrome (VLBWIs with RDS) in Japan and clarify the current status and challenges of this intervention.

**Methods:**

This observational study analysed nationwide administrative data associated with the diagnostic procedure combination system in Japan (2014–2019 fiscal year). A total of 16 429 VLBWIs with RDS were included.

**Results:**

The overall rate of pulmonary physical rehabilitation during hospitalisation was 4.1%. Infants who received rehabilitation were more likely to have been born at a gestational age of < 28 weeks and had a birth weight of < 1000 g (*p* < 0.001). The majority of pulmonary physical rehabilitation interventions lasted 20 min. On average, rehabilitation was initiated approximately 30 days after birth.

**Interpretation:**

The results of this study indicate that pulmonary physical rehabilitation performed during hospitalisation for VLBWIs with RDS is not widespread in Japan. The number of days to intervention may be a key indicator for optimising future pulmonary physical therapy interventions for VLBWIs with RDS.

**Summary**

- Japan's implementation rate of general rehabilitation interventions for VLBWIs by rehabilitation professionals of below 10% is low.
- Pulmonary rehabilitation implementation rate was only 4.1% in VLBWIs, lower among ELBWIs with RDS at 2.6%, and lowest in extremely preterm infants at 2.5%.
- The number of days to pulmonary rehabilitation, typically initiated approximately 30 days postpartum, may be a key indicator for optimising future pulmonary physical therapy interventions for VLBWIs with RDS.

## 1 Introduction

Recent advances in medical technology have improved the survival rate of very low‐birth‐weight infants (VLBWIs). According to the Japanese Vital Statistics, of approximately 890 000 newborns born in 2019, 9.4% were VLBWIs. Among them, the approximately 65 000 (7.3%) VLBWIs were managed in neonatal intensive care units (NICUs) [1]. Japan is reported to have one of the best perinatal care systems globally and among the lowest infant mortality rates for VLBWIs [1, 2].

VLBW is often present with severe respiratory problems, with nearly half developing neonatal respiratory distress syndrome (RDS), and are at high risk of chronic lung disease (CLD) [3]. CLD has been associated with impaired brain development due to hypoxia and altered respiratory‐circulatory dynamics, leading to developmental delay during infancy and school age. It is also associated with lower intelligence quotients in school‐aged children [4]. In addition, CLD diagnosed at 36 weeks is associated with increased hospitalisation for respiratory‐related illnesses, decreased respiratory function and poor neurodevelopmental prognosis after discharge. In the chronic phase, it may be complicated by pulmonary hypertension, which increases the risk of death [5]. Therefore, early ventilator weaning and prevention of reintubation and severe CLD are important issues in neonatal care.

Therefore, the ‘Guidelines for Respiratory Physiotherapy in the NICU’ were established in Japan, recommending early pulmonary physical rehabilitation intervention for neonatal respiratory disorders [6]. The main objectives of pulmonary physical rehabilitation in neonates are to prevent and improve alveolar collapse (ventilation promotion), prevent and improve secretion retention (expectoration promotion) and maintain or improve oxygenation. These guidelines, along with other sources, also mention the effectiveness and risks of these methods [7, 8, 9, 10, 11, 12]. However, the implementation rate of general rehabilitation interventions for VLBWIs by rehabilitation professionals (physical therapists [PTs], occupational therapists [OTs] and speech therapists [STs]) in Japan is low, reported at below 10% [3]. Furthermore, there are no clear data on the implementation rate and number of days from birth to intervention, from inpatient to pulmonary physical rehabilitation, for VLBWIs with respiratory diseases. Globally, reports on the implementation and effectiveness of pulmonary physical rehabilitation in neonates are limited. Given that approximately 50% of VLBWIs develop respiratory disorders, there is some guidance on appropriate pulmonary physical rehabilitation strategies. However, clear evidence is lacking regarding interventions performed by rehabilitation professionals during the hospitalisation of VLBWIs with respiratory disorders. It is possible that appropriate rehabilitation is not being provided to VLBWIs requiring pulmonary physical rehabilitation. It is hoped that clarifying this may facilitate the consideration of rehabilitation interventions for VLBWIs requiring pulmonary physical rehabilitation.

In the present study, we aimed to assess the implementation rate and number of days from birth to pulmonary physical rehabilitation intervention during hospitalisation for VLBWIs whose condition is complicated by RDS (VLBWIs with RDS) in Japan, using nationwide real‐world data. We also discuss the current global paucity of evidence on the implementation and effectiveness of pulmonary physical rehabilitation interventions in neonates.

## 2 Materials and Methods

### 2.1 Study Design and Data Source

This observational study used data from a nationwide Japanese administrative database; the diagnosis procedure combination (DPC) system. The DPC, a case‐mix patient classification system initiated by the Ministry of Health, Labour and Welfare of Japan in 2002, provides an approach for calculating reimbursement by combining a comprehensive evaluation. This evaluation consists of a fixed number of points per day, as determined by the Ministry of Health, Labour and Welfare, and a piece‐rate evaluation based on medical procedures, such as surgery, and patient illness. The data included hospital location, admission date, patient age and sex, diagnoses, complications at and after admission, procedures, medications and equipment used, length of stay, in‐hospital mortality and pregnancy status [13]. In the DPC database, diagnoses are recorded using the International Classification of Diseases, 10th revision (ICD‐10) codes, with text data entered in Japanese. DPC data were collected by the DPC Study Group and cover approximately 90% of tertiary emergency hospitals in Japan [7]. DPC data can be used to analyse the utilisation, access, outcomes and costs of medical services [8]. Clinical research using this database has increased in recent years [9].

In the present study, we employed a retrospective observational design using the DPC data. The study design was approved by the appropriate Ethics Committee for Medical Research, which waived the requirement of informed consent. The study is reported following the Strengthening the Reporting of Observational Studies in Epidemiology (STROBE) guidelines, with a completed checklist submitted.

### 2.2 Pulmonary Physical Rehabilitation During Hospitalisation

Patients whose medical fees were claimed for pulmonary physical rehabilitation had received pulmonary physical rehabilitation from rehabilitation professionals during hospitalisation.

### 2.3 Other Variables

Sex, gestational age, birth weight, year of hospitalisation, discharge destination, length of hospital stays and duration of pulmonary physical rehabilitation were identified. The infants were categorised as extremely preterm (< 28 weeks), very preterm (28–31 weeks), moderately preterm (32–33 weeks), late preterm (34–36 weeks), or full‐term/late term/post‐matter/post‐mature (> 36 weeks) according to their gestational age. The infants were also classified into extremely‐low‐birth‐weight infants (ELBWIs, ≤ 999 g) and VLBWIs (1000–1499 g) categories, according to birth weight. Comorbid RDS was identified based on the ICD‐10 code, P220.

### 2.4 Japan's Physical Rehabilitation Delivery System

In Japan, ‘disease‐specific rehabilitation fees’ are calculated for physical rehabilitation services by rehabilitation professionals. The disease‐specific rehabilitation fee is a reimbursement system that allows facilities to calculate healthcare fees when providing physical rehabilitation to patients and is divided into five disease categories: cerebrovascular, musculoskeletal, respiratory, cardiovascular and disuse‐related rehabilitation. To investigate pulmonary physical rehabilitation for VLBWIs with RDS, we decided to limit the classification calculation to the respiratory rehabilitation fee.

The fee is calculated when rehabilitation is provided to a patient as individual therapy for ≥ 20 min. The calculation method follows a standardised system, where one unit corresponds to 20 min of physical rehabilitation (the same calculation method is used for all disease‐specific rehabilitation fees). Additional units are counted in 20‐min increments.

### 2.5 Statistical Analysis

The infants were classified into two groups based on whether they received inpatient pulmonary physical rehabilitation. These groups were compared in terms of sex, gestational age, birth weight, year of hospitalisation, length of hospital stay and discharge destination.

Infants who were rehabilitated during hospitalisation were classified into two groups: VLBWIs with RDS or ELBWIs whose condition was complicated by RDS (ELBWIs with RDS), and the number of days from admission to the start of rehabilitation, the number of days of rehabilitation during hospitalisation, hours of rehabilitation per day, and hours of rehabilitation per day during the hospitalisation period were compared between the two groups.

Categorical variables were compared between the two groups using the chi‐squared test, and continuous variables were compared using the Mann–Whitney U test. All statistical analyses were performed using Stata Statistical Software Release 18 (Stata Corp LLC, College Station, TX, USA). Statistical significance was set at *p* < 0.05.

## 3 Results

**FIGURE 1:**
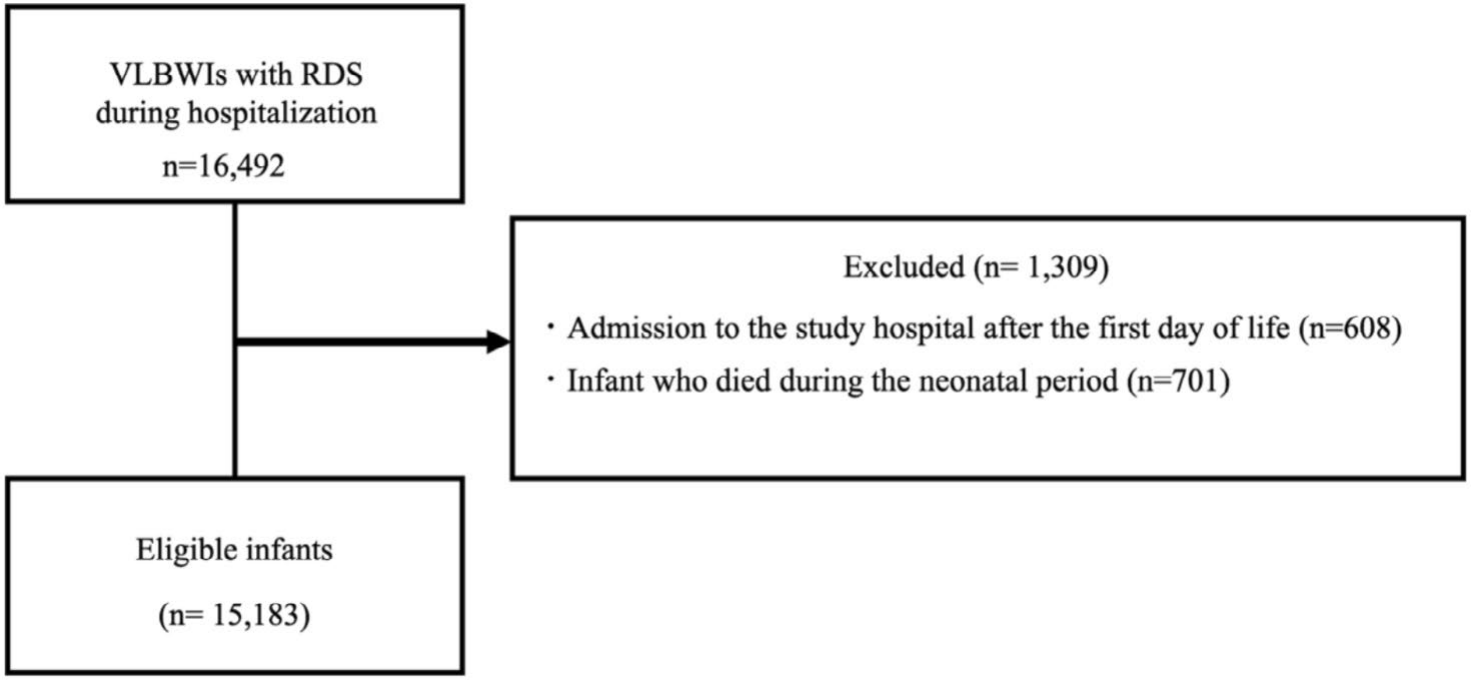
Flowchart for the inclusion of patients in the present study. RDS, respiratory distress syndrome; VLBWI, very low‐birth‐weight infant.

We identified VLBWIs with neonatal RDS (ICD‐10 code: P220) who were discharged from eligible hospitals between January 2014 and March 2020 (*n* = 16 492). Exclusion criteria were admission to the target hospital after the first day of life (*n* = 608) and neonatal death (*n* = 701). Finally, 15 183 infants were included in the analysis (Figure 1).

Table 1 presents the patient characteristics according to the pulmonary physical rehabilitation status. A higher percentage of infants who received pulmonary physical rehabilitation had a gestational age of < 28 weeks and had extremely low body weights than those who did not receive pulmonary physical rehabilitation (*p* < 0.001).

**TABLE 1:** Characteristics of the participants (*n* = 15 183).

Characteristics	During hospitalisation		*p* *
	With pulmonary rehabilitation (*n* = 628)	Without pulmonary rehabilitation (*n* = 14 555)	
Sex			0.41
Male	319 (50.8%)	7639 (52.5%)	
Female	309 (49.2%)	6916 (47.5%)	
Gestational age (weeks)			< 0.001
Extremely preterm (< 28 week)	374 (50.8%)	6821 (46.9%)	
Very preterm (28–31 week)	221 (49.2%)	6769 (46.6%)	
Moderate preterm, late preterm, full‐term/late‐term/post‐term/post‐mature (≥ 31 week)	32 (5.1%)	942 (6.5%)	
Weight (g)			< 0.001
< 1000	398 (63.4%)	7768 (53.4%)	
1000–1499	230 (36.6%)	6787 (46.6%)	
Fiscal year			0.003
2014	88 (14.0%)	2634 (18.1%)	
2015	101 (16.1%)	2496 (17.1%)	
2016	93 (14.8%)	2409 (16.6%)	
2017	97 (15.4%)	2329 (16.0%)	
2018	123 (19.6%)	2421 (16.6%)	
2019	126 (20.1%)	2266 (15.6%)	

* Chi‐squared test.

Table 2 shows the length of hospital stay and discharge destination by pulmonary physical rehabilitation status. The median hospital stay was longer in the rehabilitation group (108 days, interquartile range [IQR]: 80.5–144.0) than in the non‐rehabilitation group (90 days, IQR: 66.0–123.0) (*p* < 0.001). Additionally, infants who received pulmonary physical rehabilitation were less likely to be transferred to another hospital than those who did not.

**TABLE 2:** Outcomes of the participants (*n* = 15 183).

Characteristics	During hospitalisation		*p* *
	With pulmonary rehabilitation (*n* = 628)	Without pulmonary rehabilitation (*n* = 14 555)	
Length of stay (day), median (IQR)			
In hospital	108 (80.5–144.0)	90.0 (66.0–123.0)	< 0.001
Discharge destination			< 0.001
Home	527 (83.9%)	12 189 (83.7%)	
Other hospital/nursing home/others	65 (10.4%)	2022 (13.8%)	

Abbreviation: IQR, interquartile range.

* Chi‐squared test.

Table 3 presents the number of days from birth to intervention of pulmonary physical rehabilitation and the amount of pulmonary physical rehabilitation provided by birth weight for the 628 patients who underwent pulmonary physical rehabilitation during hospitalisation. The ELBWI group had a longer time from admission to the number of days from birth to intervention of pulmonary physical rehabilitation (median: 35.0 vs. 20.0 days, *p* < 0.001) than the VLBWI group.

**TABLE 3:** Information of participants (*n* = 628).

Characteristics	Weight (g)		*p* *
	< 1000 (*n* = 398)	1000–1499 (*n* = 230)	
Number of days related to pulmonary physical rehabilitation intervention			
Number of days from birth to intervention, median (IQR)	35.0 (17.0–71.0)	20.0 (9.0–42.0)	< 0.001
Number of days in hospital, median (IQR)	32.0 (13.0–51.0)	25.0 (11.0–39.0)	< 0.001
Time associated with pulmonary physical rehabilitation interventions			
Intervention time (min/days)			0.021
20	295 (74.1%)	189 (82.2%)	
≥ 40	103 (25.9%)	41 (17.8%)	
Intervention time/in‐hospital days			0.006
< 20	300 (75.4%)	150 (65.2%)	
≥ 20	98 (24.6%)	80 (34.8%)	

Abbreviation: IQR, interquartile range.

* Chi‐squared test.

Similarly, the number of days of inpatient pulmonary physical rehabilitation was also higher for the ELBWI group (32.0 days, IQR: 13.0–51.0) than for the VLBWI group (25.0 days, IQR: 11.0–39.0) (*p* < 0.001). The percentage of patients who received > 40 min of pulmonary physical rehabilitation per rehabilitation intervention day was also higher in the ELBWI group (103 infants [25.9%]) than in the VLBWI group (41 infants [17.8%]) (*p* = 0.021). However, the percentage of patients who received > 20 min of pulmonary physical rehabilitation per hospital stay was lower in the ELBWI group (98 infants [24.6%]) than in the VLBWI group (80 infants [34.8%]) (*p* = 0.006).

**FIGURE 2:**
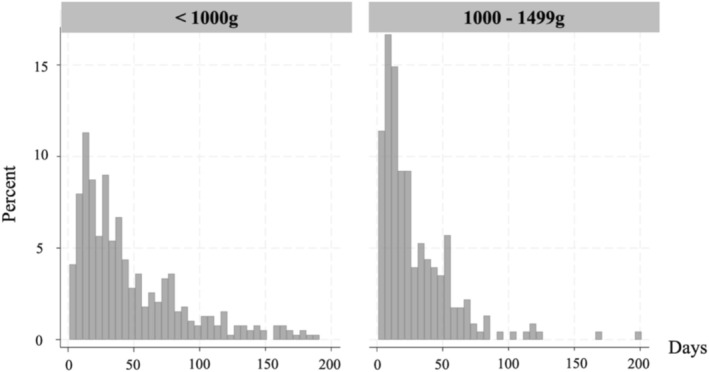
The number of days from birth to the initiation of pulmonary physical rehabilitation intervention, stratified by birth weight (ELBWIs and VLBWIs). Excluding those for whom the number of days from birth to the pulmonary physical rehabilitation intervention exceeded 200 days (*n* = 11). ELBWI, extremely low birth weight infant; VLBWI, very low‐birth‐weight infant.

Although both the ELBWI and VLBWI groups had a similar percentage of pulmonary physical rehabilitation interventions within 100 days of birth, the VLBWI group had a higher percentage of pulmonary physical rehabilitation interventions within 50 days of birth (Figure 2).

## 4 Discussion

In the present study, we investigated the implementation rate, timing and outcomes of pulmonary physical rehabilitation for VLBWIs with RDS using the Japanese DPC database. The study revealed that the intervention rate of pulmonary physical rehabilitation by rehabilitation professionals for VLBWIs with RDS was low, with pulmonary physical rehabilitation initiated at approximately 30 days after birth.

The strength of our study lies in its analysis of a large sample from the Japanese DPC database, covering the admission of 15 183 VLBWIs with RDS over the past 6 years. The DPC data we used cover 90% of tertiary emergency hospitals; therefore, the results of our study are considered largely reflective of the current situation in Japan [13].

The study revealed a low intervention rate of pulmonary physical rehabilitation for VLBWIs with RDS, at only 4.1%. In particular, it was even lower in ELBWIs with RDS (2.6%) and extremely preterm infants (2.5%). Two factors may contribute to the low implementation rate. First, the limited availability of rehabilitation professionals in NICUs in Japan [10]. Second, the importance of pulmonary physical rehabilitation in neonatal care is not fully recognized [10, 11], and this may be partly explained by the lack of an established rehabilitation curriculum for neonates. To improve the implementation rate of pulmonary physical rehabilitation for VLBWIs with RDS in the future, in addition to the training and placement of rehabilitation professionals specialising in neonatal respiratory disorders, it is necessary to educate healthcare professionals in general.

Very preterm infants and ELBWIs with RDS were more in the intervention group than among infants who did not receive pulmonary physical rehabilitation. This suggests that pulmonary physical rehabilitation is provided to more severely affected infants. Extremely preterm infants and ELBWIs with RDS are known to be at a higher risk of developing secondary conditions, such as patent ductus arteriosus and necrotising enterocolitis. Additionally, prevention of early ventilator‐based extubation and reintubation is important in these infants. The results of this study suggest that there is a need to increase the implementation rate of pulmonary physical therapy for VLBWIs with RDS in Japan, to prevent respiratory‐related diseases, improve respiratory function and reduce the severity of CLD.

The duration of most pulmonary physical rehabilitation interventions for VLBWIs with RDS was 20 min. For physical rehabilitation of VLBWIs in general, a 20‐min intervention has been reported to be appropriate [12]. Similar intervention times have also been reported in neonatal pulmonary physical rehabilitation without adverse events [14]. While the optimal duration for pulmonary physical rehabilitation interventions in VLBWIs with RDS remains unclear, the results of the present study indicate that a 20‐min intervention is appropriate, similar to neonatal physical rehabilitation for other calculation categories.

Our study has a few limitations. First, the DPC data did not provide detailed information about the types of pulmonary physical rehabilitation performed. Therefore, our study could not include the pulmonary physical rehabilitation intervention types. However, as the Japanese guidelines recommend the promotion of ventilation, expectoration and improvement of oxygenation [6], interventions were likely provided according to the respiratory dysfunction of the VLBWIs with RDS. Second, we examined pulmonary physical rehabilitation implementation rates based on the medical claims information billing by rehabilitation professionals. Therefore, in the present study, we did not consider the pulmonary physical rehabilitation performed by other professionals, including nurses, and the implementation rate may have been underestimated. Finally, the study did not clarify the factors contributing to the low implementation rate of pulmonary physical rehabilitation, making our interpretation speculative. Future studies should examine the factors associated with the low implementation rates of pulmonary physical rehabilitation in VLBWIs with RDS.

In summary, in the present study, we analysed a large sample from the Japanese DPC database and found low levels of pulmonary physical rehabilitation interventions for VLBWIs with RDS and the timing of intervention initiation. In particular, the days from birth to intervention of pulmonary physical rehabilitation for VLBWIs with RDS were found to be approximately 30 days after birth, which is novel. The results of the present study provide a basis for improving the implementation rate of pulmonary physical rehabilitation in the future treatment of VLBWIs with RDS.

## 5 Conclusions

This 6‐year observational study using DPC data found that pulmonary physical rehabilitation was provided to only 4.1% of hospitalised VLBWIs with RDS. The implementation rate was particularly low for ELBWIs with RDS (2.6%) and infants with a gestational age < 28 weeks (2.5%), highlighting the rarity of interventions for unstable neonatal conditions. Additionally, pulmonary physical rehabilitation was typically initiated approximately 30 days after birth for VLBWIs with RDS. These findings underscore the limited implementation of pulmonary physical rehabilitation for VLBWIs with RDS in Japan. The results of this study provide a basis for improving the implementation rate of pulmonary physical rehabilitation in the future treatment of VLBWIs with RDS.

## Author Contributions

**Yuto Ogata:** conceptualization, methodology, writing – original draft, visualization. **Ryutaro Matsugaki:** conceptualization, methodology, formal analysis, writing – review and editing, project administration. **Manami Zaizen:** writing – review and editing. **Keiji Muramatu:** investigation, writing – review and editing. **Shinya Matsuda:** investigation, data curation, writing – review and editing, funding acquisition. **Shutaro Suga:** conceptualization, writing – review and editing. **Satoru Saeki:** writing – review and editing, supervision, funding acquisition. All authors have read and agree with the published version of the manuscript.

## Ethics Statement

The present study was conducted in accordance with the Declaration of Helsinki and approved by the Institutional Review Board of the University of Occupational and Environmental Health (protocol code R4‐045).

## Consent

The Ethics Committee for Medical Research at the University of Occupational and Environmental Health waived the requirement for informed consent.

## Conflicts of Interest

The authors declare no conflicts of interest.

## Data Availability

The data that support the findings of this study are openly available in YUTO OGATA at https://pubmed.ncbi.nlm.nih.gov.
